# Premature death associated with long-term evacuation among a vulnerable population after the Fukushima nuclear disaster: A case report

**DOI:** 10.1097/MD.0000000000016162

**Published:** 2019-07-05

**Authors:** Toyoaki Sawano, Yoshitaka Nishikawa, Akihiko Ozaki, Claire Leppold, Mai Takiguchi, Hiroaki Saito, Yuki Shimada, Tomohiro Morita, Manabu Tsukada, Hiromichi Ohira, Masaharu Tsubokura

**Affiliations:** a Department of Surgery, Minamisoma Municipal General Hospital; b Department of Public Health, Fukushima Medical University School of Medicine; c Department of Internal Medicine, Soma Central Hospital, Fukushima; d Department of Health Informatics, School of Public Health, Kyoto University, Kyoto; e Department of Breast Surgery, Jyoban Hospital of Tokiwa Foundation; f Research Center for Community Health, Minamisoma Municipal General Hospital, Fukushima, Japan; g Global Public Health Unit, School of Social and Political Science, University of Edinburgh, Edinburgh, UK.; h Department of Cardiology, Sukagawa Hospital, Fukushima; i Department of Gastroenterology, Sendai Kousei Hospital, Miyagi; j Department of Neurosurgery, Minamisoma Municipal General Hospital, Fukushima, Japan.

**Keywords:** fukushima daiichi nuclear power plant, fukushima nuclear disaster, individual with physical disability, long-term evacuation, temporary houses, the great east japan earthquake and tsunami, vulnerability

## Abstract

**Rationale:**

The health vulnerability of certain populations such as children, the elderly and individuals with illnesses or physical disability can become significant in disasters. After the 2011 Fukushima Daiichi Nuclear Power Plant (FDNPP) accident, significant health impacts on vulnerable populations were observed during early or mid-term phase of the disaster, presumably associated with the evacuation. However, there is limited information available on the health impacts owing to long-term evacuation after disaster among them.

**Patient concerns:**

A 56-year-old physically challenged male with arteriovenous malformation on his right lower limb, diagnosed when he was 2 years’ old, lived near the FDNPP. He and his family were forced to evacuate immediately after the accident.

**Diagnosis:**

Three months after evacuation following the FDNPP accident, he developed a refractory foot ulcer associated with atrial fibrillation and congestive cardiac failure because of deterioration of arteriovenous malformation, presumably led by repeated evacuations.

**Intervention:**

Although anticoagulation therapy and diuretic therapy improved his cardiac failure in the initial admission, he decided to only be treated with supportive care after revelation that his arteriovenous malformation was no longer eligible for aggressive intervention.

**Outcome:**

Three years after the long-term evacuation in temporary houses, the patient died of bleeding and infection of the ulcer.

**Lessons:**

This case suggests that long-term evacuation for individuals with physical disability may lead to significant health impacts, and even premature death, through the deterioration of daily life activities due to physical and psychological burdens. This case presents a need for further research on ways that disasters impact the health of individuals with physical disabilities, and greater disaster preparation for the needs of populations with physical disabilities.

## 1 Introduction

The health vulnerability of certain populations can become profound in disasters.^[1]^ Children, pregnant women, the elderly, individuals with illnesses or physical disability and those with low socioeconomic status such as migrant workers are particularly vulnerable when a disaster occurs.^[1–6]^ Until now, the majority of health impacts among vulnerable populations have been observed during the early or mid-term (weeks to months) phase after disasters, such as hurricanes, floods, and earthquakes.^[3,7,8]^ However, little information is available on long-term (years) health risks after a major disaster, which could be important public health and policy issue, given that disaster recovery can span during a long period.

After the 2011 Fukushima Daiichi Nuclear Power Plant (FDNPP) accident, significant health impacts on vulnerable populations, such as the elderly and hospitalized patients, were observed during early or mid-term phase of the disaster, presumably associated with the evacuation.^[9–12]^ The Japanese government issued mandatory evacuation orders to areas surrounding the FDNPP, and the evacuation orders continue in areas closest to the FDNPP as of March 11, 2018.^[11]^ It has previously been suggested that nuclear disasters may have a longer impact than other disasters such as earthquakes and tsunamis^[13]^; however, there is limited information available on the full range of potential health impacts owing to long-term evacuation after disaster among vulnerable population.

We experienced a case of a 56-year-old man with a arteriovenous malformation of his right lower limb, of which the prognosis was considered to be significantly influenced by prolonged mandatory evacuation after the FDNPP accident. Assessment of this case, and the possible ways that evacuation may have impacted individuals with physical disabilities, may be helpful to understand ways to limit the health impacts of long-term evacuation on vulnerable populations in future disasters.

## 2 Case presentation

**Figure 1:**
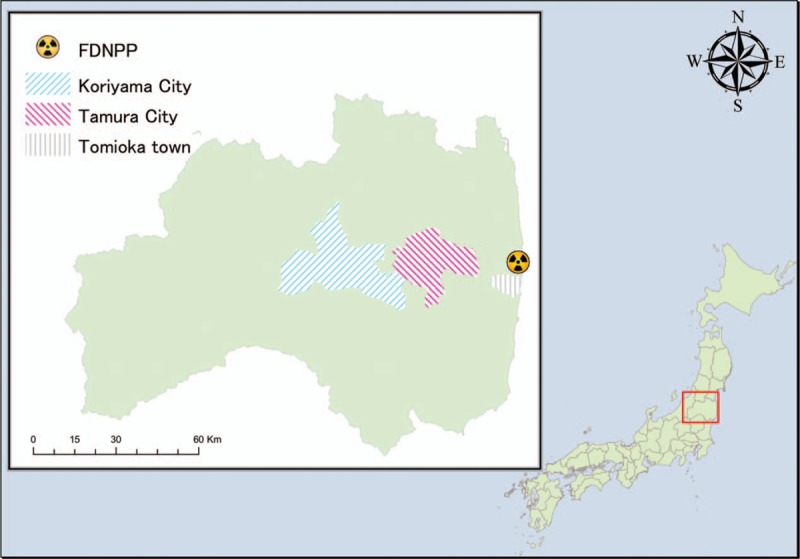
Tomioka Town is located 5 to 14 km south from the Fukushima Daiichi Nuclear Power Plant. Tamura City is 40 km west of Tomioka Town. Koriyama city is located in 60 km west from Tomioka town.

A 56-year-old male with a history of right lower limb arteriovenous malformation, diagnosed when he was 2 years’ old, was living in Tomioka Town, located 5 to 14 kilometers south from the FDNPP, before the FDNPP accident (Fig. 1). He was recognized as having a grade 3 extremity disability (a unilateral lower limb that does not function completely) by the Act for the Welfare of Persons with Physical Disabilities in Japan. Although his right lower limb was difficult to use, he could walk using crutches. Some support was necessary from his family members; however, he had been able to live without limitations in daily activities before the disaster.

On March 11 in 2011, the patient experienced the Great East Japan Earthquake while at his home with his family. The tsunami did not reach their house. The next day, the Japanese central government declared Tomioka town as part of the mandatory evacuation zone. The patient and his family immediately evacuated to an evacuation center in Tamura city, 40 km west from their home. (Fig. 1) Although the evacuation center was open to all evacuees, no space was particularly prepared for people with physical disabilities, leading our patient to spend almost all of his time in his car because he felt that his existence in the public space may become a nuisance to others. Several days later, he moved to a room in a hotel in Koriyama city, 60 km west from Tomioka town. (Fig. 1) Dramatic environmental changes from repeated evacuations imposed a significant physical burden to our patient. He also experienced psychological stress, feeling that he was imposing a strain on family because he thought that his existence prevented his family from being able to freely evacuate.

Three months after initial evacuation, he was referred to a hospital near the hotel in Koriyama city, with symptoms of fever and palpitation. He was admitted to the hospital with a new diagnosis of atrial fibrillation and congestive cardiac failure. During this admission there was deterioration of his right lower limb arteriovenous malformation, located between his abdominal aorta and right femoral artery, which caused a right foot ulcer. He was treated with anticoagulation therapy and diuretic therapy, and was discharged from the hospital approximately one month after the admission, although his foot ulcer required frequent care because it was refractory after hospital discharge.

The hospital admission after repeated evacuations notably weakened our patient's physical activity. After hospital discharge (approximately 4 months after his initial evacuation from Tomioka town), he moved into a temporary house constructed for evacuees in Koriyama city because the mandatory evacuation order continued. Although he could not ambulate, dress, or bathe himself without support, he did not need support for eating and using the toilet. The temporary house presented a particularly difficult environment for someone with a physical disability; unpaved roads around the house severely limited his opportunities to go outside and the bath was too narrow to use while requiring support from family.

His physical condition gradually worsened. Continuation of frequent care, such as washing and applying ointments, did not improve his foot ulcer. Following a detailed medical examination in February 2012, which revealed that his arteriovenous malformation was no longer eligible for aggressive intervention, he decided to only be treated with supportive therapy. From August 2012, he became confined to bed and required assistance for all activities of daily living. In July 2013, he was no longer able to go out to temporarily visit his original home in the evacuation zone, which had been only thing he looked forward to. He prioritized time with his family, did not like being admitted to hospital, and did not seek medical care outside of periodic visits to the clinic. Although he was hardly eating, and was aware of pain in his right limb and shortness of breath from December 2013, he did not visit the hospital. In the middle of a night in February 2014, he was transported to the hospital after massive bleeding from his right foot ulcer. After admission to the hospital, infection to the ulcer was found in addition to refractory bleeding. Despite blood infusion therapy and antibiotic therapy, he died of septic shock 18 days after admission.

## 3 Discussion

In the present case, a 56-year-old physically challenged male with arteriovenous malformation on his right lower limb developed a refractory foot ulcer associated with atrial fibrillation and congestive cardiac failure due to deterioration of arteriovenous malformation soon after evacuation following the FDNPP accident. This led to his death 3 years later as a consequence of bleeding and infection of the ulcer. This case suggests that long-term evacuation in facilities with improper accommodation for individuals with physical handicaps may lead to significant health impacts, and even premature death, through the deterioration of daily life activities because of physical and psychological burdens.

In this case, although the patient died of infection and bleeding in his refractory foot ulcer, the ulcer was essentially caused by deterioration of underlying arteriovenous malformation in his right lower limb after repeated evacuations. External irritation such as repeated translocation may have exacerbated his arteriovenous malformation.^[14,15]^ After the Fukushima disaster, the risk of death after immediate evacuation among residents of an elderly care facility, another vulnerable population, was found to have been about 3.4 times higher than those who did not immediately evacuate.^[10]^ The present case indicates that mandatory evacuation may have contributed to the risk of premature death among individuals with physical disabilities, in addition to the elderly. It has been reported that disaster-related deaths occurred after the nuclear disaster and lasted for a prolonged period, in contrast to direct deaths from the earthquake and tsunami.^[13]^ It is therefore possible that mortality among vulnerable populations has increased over a long period, even outside of this case. Further analysis is necessary to understand the full extent of disaster impacts on vulnerable populations such as those with physical disabilities, and to elucidate the types of support that can mitigate disaster burdens to this population, to improve their prognosis in disaster settings.

Vulnerable populations such as those with physical disabilities may also be more sensitive to environmental and psychological burdens after a disaster, compared to the general population. In this case, the feeling of imposing a strain on his family during evacuation, and the inadequate preparation for individuals with physical disabilities in governmental evacuation centers and temporary housing, may have played important roles in the health deterioration of this patient. The environment during the prolonged period in temporary housing, which was not adapted for those with physical disabilities, may have worsened his health, and is indicative of a policy area where the Japanese government must improve disaster preparation to include individuals with physical disabilities.

In conclusion, long-term evacuation after the FDNPP disaster contributed to health deterioration and premature death in an individual with a physical disability. This case presents a need for further research on ways that disasters impact the health of individuals with physical disabilities, and greater disaster preparation and response to account for the needs of populations with physical disabilities in Japan.

## Acknowledgments

The authors thank the family of the patient for providing the detailed patient's information through an interview, and also thank Masatsugu Tanaki for his constructive opinions on this study.

## Author contributions

Sawano T, Nishikawa Y, Ozaki A, Morita T and Tsubokura M contributed to the conception and design of the research. Sawano T drafted the article. All the authors performed the critical revision of the article for important intellectual content, and involved in the interpretation of the case and final approval of the article.

**Conceptualization:** Yoshitaka Nishikawa, Akihiko Ozaki, Claire Leppold, Tomohiro Morita, Masaharu Tsubokura.

**Supervision:** Yoshitaka Nishikawa, Akihiko Ozaki, Claire Leppold, Mai Takiguchi, Hiroaki Saito, Yuki Shimada, Tomohiro Morita, Manabu Tsukada, Hiromichi Ohira, Masaharu Tsubokura.

**Writing – original draft:** Toyoaki Sawano.

**Writing – review & editing:** Claire Leppold, Masaharu Tsubokura.

Toyoaki Sawano orcid: 0000-0002-1482-6618.
